# Masticatory Muscles Activity in Sport Climbers

**DOI:** 10.3390/ijerph17041378

**Published:** 2020-02-21

**Authors:** Michał Ginszt, Grzegorz Zieliński, Aleksandra Byś, Piotr Gawda, Piotr Majcher

**Affiliations:** 1 Department of Rehabilitation and Physiotherapy, Medical University of Lublin, 20-059 Lublin, Poland; piotr.majcher@umlub.pl; 2 Department of Sports Medicine, Medical University of Lublin, 20-059 Lublin, Poland; grzegorz.zielinski.um@gmail.com (G.Z.); bysaleksandra@gmail.com (A.B.); piotr.gawda@umlub.pl (P.G.)

**Keywords:** electromyography, sport climbing, temporalis anterior, masseter muscle

## Abstract

Masticatory muscle activity during teeth clenching is associated with changes in many physiological parameters throughout the body. Clenching can improve muscle activity, force production, rate of force development, and joint fixation. Hence, teeth clenching and masticatory muscle activity can be important in competitive sports activities. Sport climbing is becoming increasingly popular and will be included for the first time in the Summer Olympic Games, Tokyo, 2020. However, masticatory muscle activity in sport climbers has not yet been studied. The aim of the presented study is to compare the bioelectrical activity of the masticatory muscles in sport climbers and non-climbers in order to determine the relationship between these muscles and climbing activity. 44 subjects without masticatory system disorders (16 women and 28 men, average age 26.3) were divided into two groups of 22 sport climbers (8 women, 14 men, climbing experience >4 years), while 22 people (8 women, 14 men, with no regular sports activity) were assigned to the control group. Electromyographic examination of temporalis anterior (TA) and masseter muscle (MM) was evaluated in three conditions: during resting mandibular position, during maximum intercuspation clenching, and during maximum voluntary clenching with cotton rolls between teeth. For statistical analysis, the W Shapiro-Wilk test and the Mann-Whitney U test were used. Sport climbers showed significantly higher bioelectrical activities of MM during maximum intercuspation clenching (238.45 μV vs. 83.87 μV, *p* = 0.002), and during maximum voluntary clenching with cotton rolls between teeth (300.01 μV vs. 101.38 μV, *p* = 0.001) compared to controls. The differences between groups in relation to the resting bioelectrical activity of the MM muscles, and TA muscles in all conditions were not statistically significant (*p* > 0.05). Higher bioelectrical activity of masseter muscles during clenching in climbers can be associated with this sports activity. However, the mechanism remains unknown and requires future research.

## 1. Introduction

Over the past 40 years, there has been a growing interest in extreme sports, and the number of extreme athletes is starting to outweigh the number of participants in traditional sports [1]. The definition of extreme sports includes, among others, sport climbing [2]. This discipline is considered both as recreational physical activity and competitive sport, which is associated with a wide spectrum of participants, bringing together both amateurs and professionals. In 2020, this sport will for the first time be included in the Summer Olympic Games in Tokyo [3].

The extreme sports trend is also associated with an increased number of dangerous events and injuries related to the specificity of a given sport [2,4]. In addition, regular training affects the formation of structural and functional changes in all systems of the human body. However, too much training load and lack of proper regeneration lead to the crossing of the body’s adaptive barrier and, as a result, to injury [5]. Extreme sport is also associated with a high psychological burden for athletes [4]. Knowledge of adaptive changes, psychology, pathophysiology, and epidemiology of injuries associated with practising extreme sports can provide a basis for developing diagnostic, therapeutic, and preventive treatment schemes in extreme sports medicine. Current literature indicates that adaptations of the climber’s body mainly include the myofascial and nervous systems, resulting in, among others, an increase in the isometric strength of the torso flexors and extensors and hand strength, an increase in the flexibility of the torso in the sagittal and frontal planes, and enlargement of brain structures responsible for efficient hand movements, hand-eye coordination, and correction of movement [3,6,7]. In addition, this sport can affect the psychological aspects of health, including reducing depression and anxiety and maintaining emotional stability [8]. However, sport climbing like any extreme sport is associated with a high mental load on the athlete, in particular high exposure to stress, which can lead to the development of temporomandibular disorders (TMDs) and masticatory muscle pain [9,10].

On the other hand, teeth clenching can cause an increase in some physical parameters, such as, e.g., muscle activity and force production [11]. This fact may suggest that in athletes practicing strength sports changes can be observed in the bioelectric potentials of the masticatory muscles. Moreover, the periodontal mechanoreceptors determine all the functional physiological and pathological processes of the stomatognathic system, playing an important role in the adaptive and compensatory sensorimotor cortical processes [12,13]. Therefore, the role of masticatory muscles and periodontal mechanoreceptors in muscle activity and force production is relevant in the context of athletic performance.

However, current literature lacks research on adaptive changes within the stomatognathic system occurring in sport climbers. Hence, the purpose of this work is to analyze masticatory muscle activity in sport climbers compared to non-climbers in order to determine the relationship between these muscles and climbing activity.

## 2. Materials and methods

### 2.1. Study Design

The tests were carried out according to the recommendations of the Helsinki Declaration and with the consent of the Bioethics Committee of the Medical University of Lublin (KE-0254/73/2017). The surveyed people were informed about the objectives of the study and were aware of the possibility of resigning at any time. All examined persons have given written consent for the above-mentioned research. All tests were performed in the Department of Functional Masticatory Disorders, Medical University of Lublin, by experienced researchers, dentists, and physiotherapists.

### 2.2. Participants

44 subjects without masticatory system disorders (16 women and 28 men, average age 26,3) were divided into 2 groups of 22 sport climbers (8 women, 14 men, climbing experience > 4 years), while 22 people (8 women, 14 men with no regular sports activity) were assigned to the control group. All participants were clinically examined on the basis of a two-axis Research Diagnostic Criteria for Temporomandibular Disorders (RDC/TMD) form [14]. The following exclusion criteria were used: occurrence of headache and cervical spine pain within the month preceding the examination; head and neck injuries within the last six months before the examination; previous head and neck surgical treatment within the last six months before the examination; pregnancy; craniofacial trauma; painful form of TMD found according to the RDC/TMD; subjective self-assessment of the state of health in the RDC/TMD form below sufficient; class II and III of the bite according to Angle’s classification; open bite; lack of more than four teeth within both dental arches; condition during orthodontic treatment; lack of four support zones in dental arches; possession of dental prostheses (regardless of type); mental disorders; and neurological disorders.

These inclusion criteria were applied to the study group: climbing experience longer than 2 years and the number of hours of training on the climbing wall minimum 10 h a week; the following criteria were used in the control group: no regular sport activity measured by the Polish version of the International Physical Activity Questionnaire [15].

### 2.3. Data Collection

The electromyographic tests were conducted between 9 and 11 a.m., in order to minimize the influence of daily fluctuations of masticatory muscle activity. Each participant was prepared for the test as follows: the subjects’ skin was cleaned with 90% ethyl alcohol to reduce impedance, and then the subjects sat on a dental chair. The head was based on the headrest of the chair with the torso perpendicular to the ground, with the lower limbs straightened, laid out freely and parallel. Disposable round Ag/AgCl electrodes with a diameter of 30 mm and a conductive surface of 16 mm (SORIMEX, Toruń, Poland) were glued in the above position. The arrangement of surface electrodes was always carried out by the same person (MSc in Physiotherapy), in accordance with the guidelines of the SENIAM program [16]. Two electrodes were placed on one muscle belly, symmetrical on both sides, according to the course of the fibers of the anterior temporalis muscle [TA] and the superficial part of the masseter muscle [MM]. The reference electrode was placed on the forehead [16,17]. The study used the 8-channel BioEMG IIITM surface electromyography apparatus with the BioPak Measurement System (BioResearch Associates Inc., Milwaukee, WI, USA). The data collection was performed by the trained physiotherapist with experience in electromyography measurements. Before the test, an interference test was performed to check the reliability of the surface electromyographic (sEMG) signal. The signal was recorded in three conditions: during resting mandibular position, during maximum intercuspation clenching, and during maximum voluntary clenching with cotton rolls between teeth, according to the protocol of Wieczorek et al. [17,18,19,20].

### 2.4. Statistical Analysis

The data comparison was compiled and performed using the IBM SPSS STATISTICS 21 program. First, the normality of the distribution of variables was verified using the Shapiro-Wilk test and the Kolmogorov-Smirnov test (with the Lillierfors correction). All distributions did not fulfill normal distribution, which is why the non-parametric Mann-Whitney U test was used later. The differences were considered statistically significant if the level of test probability was lower than the assumed level of significance (*p* < 0.05).

## 3. Results

Sport climbers showed significantly higher bioelectrical activities of MM during maximum intercuspation clenching (238.45 μV vs. 83.87 μV, *p* = 0.002), and during maximum voluntary clenching with cotton rolls between teeth (300.01 μV vs. 101.38 μV, *p* = 0.001) compared to controls. The differences between groups in relation to the mean bioelectrical activity of the MM muscles during resting mandibular position and TA muscles in all three conditions were not statistically significant (*p* > 0.05) as presented in Table 1.

## 4. Discussion

The results of the author’s study showed significantly higher bioelectrical activities of the masseter muscles during maximum intercuspation clenching and during maximum voluntary clenching with cotton rolls between teeth in sport climbers. However, the resting activity of the masseter muscle and the activity of the temporal muscle did not differ between the examined groups. Hence, only functional activity within the masseter muscles appears to be associated with the climbing performance. Despite the considerable amount of research into the adaptation of the muscular system of climbers to training, there are no studies on changes in the bioelectric potential of the masticatory muscles.

The results of the work of Kawakubo et al. indicate that, in people with habitual clenching of teeth, the average maximum grip strength increases by approximately 108% during this function [21]. Therefore, these studies suggest a close relationship between the stomatognathic system and the strength of upper limb muscles. Moreover, in this study, increased clamping force was associated with the activation of relevant parts of the cerebral cortex in response to the clenching of teeth [21]. Churei’s research, on the other hand, reports that the motor functions of the stomatognathic system, such as clenching teeth, not only increase the maximum gripping force, but also the speed of gripping force [11]. According to the literature on the subject, the impact of the neuromuscular activity of the stomatognathic system also affects motor control of movement and an increase in postural stability [22]. However, the current state of knowledge does not allow us to determine whether the increased bioelectrical activity in the function of teeth clenching in climbers can be caused by an attempt to increase the physical parameters necessary in sport climbing, or is only the adaptation of muscles to frequent clenching during sports training.

Psychological factors, primarily stress and anxiety, are another factor affecting teeth clenching. Mental tension causes include, among others, unconscious stretching of the body’s muscles, including the masticatory muscles, which can be seen in teeth clenching [23]. Stress is one of the factors predisposing to the development of bruxism [9,24]. Bruxism will be associated with greater bioelectrical muscle tension [25]. Clenching teeth is a way to release psychological tension or has a defensive function against stress [26]. Considering that sport climbing is considered an extreme sport, which involves the risk of serious injury or even death, dealing with mental stress is included in the climber’s training [27], Moreover, dysfunctions of the stomatognathic system can reduce sports activity. The negative impact of dental problems on training and performance was reported by several studies [28,29].

The present study has a limitation. Nowadays, ultrasound has been widely used in the evaluation of muscle quality and quantity, including the masticatory muscles [30,31]. However, this study did not employ this imaging tool for the evaluation of muscle quality. Therefore, ultrasound imaging of masticatory muscles should be included in future studies in this field.

To sum up, the increased functional bioelectrical activity of the masseter muscles may be the result of regular, unintended teeth clenching during climbing activity to improve motor control, stability, and muscle strength. However, to confirm the above hypothesis, further research should include the electromyographic examination of masticatory muscles during climbing activity.

## 5. Conclusions

Higher bioelectrical activities of masseter muscles during clenching in sport climbers can be associated with this sports activity. However, the mechanism remains unknown and requires future research in this field.

## Figures and Tables

**Table 1:** Collective comparison of bioelectric activity of temporalis anterior muscle (TA) and masseter (MM) at rest (R), during maximum voluntary clenching (VC), and during maximum voluntary clenching with cotton rolls between teeth (CR) between sport climbers (1) and controls (2).

Muscle and Function	N	Average sEMG Activity	SD	Test Z	P
TA (R) 1	22	2.38	1.74	−0.333	0.739
TA (R) 2	22	2.53	1.57		
MM (R) 1	22	2.07	1.34	−1.462	0.144
MM (R) 2	22	1.34	0.4		
TA (VC) 1	22	145.47	74.83	−0.436	0.663
TA (VC) 2	22	123.75	43.52		
MM (VC) 1	22	238.45	141.09	−3.129	0.002 *
MM (VC) 2	22	83.87	38.89		
TA (CR) 1	22	149.02	58.72	−0.590	0.555
TA (CR) 2	22	132.64	30.08		
MM (CR) 1	22	300.01	158.70	−3.410	0.001 *
MM (CR) 2	22	101.38	21.34		

* Significant difference.
